# Sloughing Esophagitis: A Not So Common Entity

**Published:** 2014-12

**Authors:** Hossein Akhondi

**Affiliations:** Georgetown University Medical Center, Washington, DC, USA

**Keywords:** Sloughing esophagitis, Esophagitis dissecans superficialis, Pemphigus and lichen planus, Eosinophillic esophagitis, Corrosive esophagitis

## Abstract

**BACKGROUND:**

Sloughing esophagitis, also known as esophagitis dissecans superficialis, is a very rare and underdiagnosed entity with unknown incidence rate. It can be associated with bullous dermatoses and medications such as central nervous system depressants and those causing esophageal injury.

**CASE REPORT:**

A 55-years-old woman was recovering from renal failure due to rhabdomyolysis when she developed dysphagia and odynophagia. Esophagogastroduodenoscopy with biopsy was performed for suspected bullous pemphigus and confirmed sloughing esophagitis. She improved with intravenous steroids.

**CONCLUSIONS:**

Sloughing Esophagitis should enter our differential diagnosis more frequently. It is mostly a benign, self-limiting process but when associated with bullous dermatoses will require steroid treatment.

## INTRODUCTION

Sloughing esophagitis or Esophagus Dissecans Superficialis (EDS) is a term given to a rare finding on endoscopy, which is characterized by sloughing of the esophageal mucosa that may be coughed up or vomited (1). The etiology of this finding is unknown, but since its first description in the literature many years ago, multiple reports have emerged linking it to various conditions. These conditions include: Pemphigus vulgaris, lichen planus, eosinophilic esophagitis, and medications such as bisphosphonates.

Patients may report chronic dysphagia or may even report vomiting tubular casts of esophageal mucosa (2, 3). Asymptomatic cases have also been reported with endoscopists first noticing white streaks on the esophagus during an evaluation for odynophagia or dysphagia (4).

## CASE REPORT

A 55-year- old woman with past medical history of diabetes mellitus, seizures and hypertension was admitted to the hospital after sustaining a seizure and fall. She was immobile on the kitchen floor for hours before being found. Her outpatient medications included valproic acid, aspirin, amlodipine, metformin and a multivitamin. Her physical exam showed a frail lady with normal cardiac and neuro exam. Her creatinine kinase level was 22,000 mg/dl, creatinine 5 mg/dl and hemoglobin 9.8 mg/dl. She was diagnosed with uncontrolled epilepsy, anemia, rhabdomyolysis and acute renal failure. Rhabdomyolysis was attributed to the fall, hypothermia and the prolonged period of immobility. She was started on intravenous hydration and kidney and muscle markers improved over a prolonged period of time.

Approximately 10 days after her admission, she complained of dysphagia and odynophagia to solids and liquids. Upper chest pain and discomfort was also present. Esophagogastroduodenoscopy (EGD) was done and revealed plaque like whitish material with linear streaking in the esophagus. There was no evidence of any strictures, gastric ulcers, esophageal ulcers and masses. Biopsy specimens were obtained. Initial histology reported the presence of an intraepithelial vesicular area, which raised the possibility of esophageal pemphigus. Specimens were then referred to a specialized laboratory and a final diagnosis of esophageal dissecans superficialis (EDS) or sloughing esophagitis was made.

She was started on intravenous Methylprednisolone and her symptoms improved after 2 days. She was then discharged from the hospital with oral prednisone and gastroenterology follow up plan but was eventually lost to follow up.

## DISCUSSION

Sloughing esophagitis was first described in 1892. It is an under-recognized and under-reported entity. It is characterized by sloughing of large patches of superficial mucosa. It is usually visible endoscopically as strips and patches of detaching mucosa although some cases are not recognized by endoscopes (5).

Although it has been reported in association with certain diseases and medication, most cases remain idiopathic and unexplained (6). Hot beverages, medication (bisphosphonate and non-steroidal anti-inflammatory drugs), heavy smoking, achalasia, skin conditions (prurigo nodularis and bullous dermatoses), esophageal iatrogenic injury (sclerotherapy, band ligation, dilatation and mediastinal radiation), celiac disease, immunosuppression and impaired mobility have all been implicated.

Pathogenesis remains unknown. It might represent a common reaction of esophageal squamous mucosa to various types of insult (physical, chemical, thermal and immunological) or a topical allergic response (7). Some advocate an ischemic injury pattern. Biopsies from confirmed cases shows sloughing and flaking of superficial squamous epithelium with occasional bullous separation of the layers, parakeratosis and varying degrees of acute or chronic inflammation (6). There is a split in the squamous epithelium where the base appears viable and the superficial layers that slough off and appear mummified.

Clinically the patients are usually older, more debilitated as based on parameters such as home O2, nursing home residence, hospitalized individuals, metastatic cancer, organ transplantation, immunosuppression and malnutrition (8). They are more likely to be on five or more medications especially the central nervous system depressants and medication associated with esophageal injury. Of all the patients, 84% are taking meds that cause dry mouth or interfere with swallowing, 77% are on 5 or more prescription meds, 65% are on central nervous system depressants, 55% are on medication that cause esophagus injury, 52% take narcotics, 35% take benzodiazepines and 29% take anti epileptics (8). Interestingly, gastroesophageal reflux disease is less prevalent in patients with sloughing esophagitis.

Although some patients have nonspecific upper GI symptoms, sloughing esophagitis is frequently asymptomatic. Globus sensation or dysphagia (32%), nausea (32%), suspicion of gastrointestinal bleed (32%), vomiting (23%), abdominal pain (23%), heartburn (16%), chest pain (13%), hematemesis (13%), odynophagia (10%) have all been reported (8). From epigastric pain and dysphagia to more dramatic presentation of vomiting casts, the presentation varies with age and sex. Male and females are both affected although the prevalence is unknown for the most part. Endoscopic findings of plaques and membranes can be similar to candida esophagitis which need to be ruled out to avoid misdiagnosis. Plaques are 42% in the distal esophagus, 23% in entire esophagus, 19% in midsection and only 8% in proximal area (8).

In spite of its sometimes dramatic presentations, sloughing esophagitis is mostly a benign condition without lasting pathology (6). Mucosal healing can be obtained through combination of acid suppression and discontinuation of the precipitating factors and medications. When associated with bullous dermatoses, its treatment also includes steroids (7) as will be discussed below.

It might be best to consider this disease in the specific setting that cause it. It can happen because of different reasons:

### 1) Esophageal Pemphigus and Pemphigoid

Pemphigus vulgaris (PV) is an autoimmune condition, bullous in nature, and frequently characterized by involvement of mucous membranes (9). PV is a rare condition, with a reported incidence of 0.75–5 cases per million per year. There is increased incidence among people of Jewish and Mediterranean descent (10). Anti-desmoglein 1 and 3 antibodies (IgG) have been implicated in PV. Mucosal lesions in PV are common with data suggesting that 48-70% of PV cases present as mucosal lesions several months prior to skin lesions. In addition, up to 90% of patients with PV have mucosal involvement (9) Involvement of the esophageal mucosa, respiratory mucosa, conjunctiva and the anogenital area has also been reported in the literature (11, 12).

A study from India in 1994 reported 72% of patients with PV had esophageal involvement. The most common symptoms of esophageal pemphigus include dysphagia, odynophagia and occasionally gastrointestinal bleeding. Hematemesis and vomiting esophageal casts occur rarely. Cesur and co-workers reported in 2006 that while oral mucosal involvement has been well established in PV, esophageal mucosal involvement is rarely reported (13). That study also showed a lower rate of esophageal involvement than initially reported by Gomi and co-workers (46.15% vs. 87.5%) (12-13). This difference could be attributed to the patient population in each study.

Sloughing esophagitis can also present in other autoimmune bullous dermatoses including pemphigoid. Pemphigoid is a group of autoimmune sub epithelial blistering diseases, sub classified into mucous membrane pemphigoid and bullous pemphigoid. Esophageal bullae or erosions occur in 8 percent of cases and symptoms are similar to those of pemphigus.

**Figure 1:**
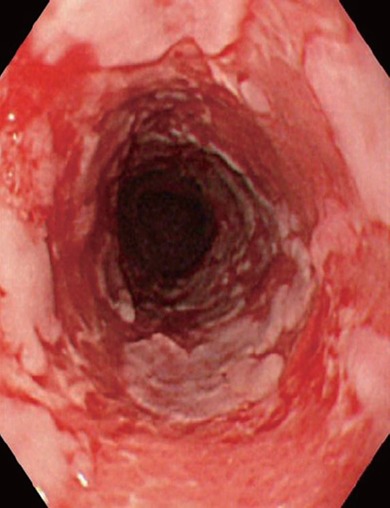
Endoscopic view of 84-year-old man with mucous membrane pemphigoid. Note esophagitis dissecans superficialis with extensive reddish erosion of the entire esophagus and whitish sheets and fragments of sloughed mucosa. Reproduced with permission from World J Gastrointest Endosc. 2010 July 16; 2(7): 252-256. Akira Hokama, MD, PhD.

Endoscopic features of sloughing esophagitis have included stripped mucosa with bleeding, total desquamation of the esophageal mucosa without bleeding, long linear mucosal break and vertical fissures and circumferential cracks with peeling, whitish mucosa with extensive bleeding and exudating esophagitis (11-13) (Fig. 1). As the esophageal mucosa is fragile with potential Nikolsky’s sign (separation of mucosa from sub mucosa) endoscopic exam should be performed carefully for correct diagnosis and prompt treatment.

Treatment of this fascinating condition depends on severity but generally involves corticosteroids and immunosuppressive agents, also acid-suppressive medications given as adjuvant treatment. It is important to note, however, that there have been reports of esophageal pemphigus developing in patients with PV after treatment with azathioprine and resolving following steroid therapy (14). Follow up with dermatology as well as gastroenterology is strongly recommended.

The long-term complications remain a mystery which can be attributed to the relatively rare occurrence of PV. Further study of esophageal pemphigus with special emphasis to these long-term complications seems appropriate.

### 2) Esophageal Lichen Planus

Lichen Planus (LP) is a mucocutaneous inflammatory condition characterized by pruritic, flat-topped, polygonal papules that commonly affect flexor skin surfaces, genitalia and other mucous membranes. In 1% of the cases the esophagus is also affected. Esophageal involvement is often asymptomatic but symptoms such as dysphagia and odynophagia have been reported, frequently secondary to strictures and esophagitis (15).

Esophagitis in esophageal lichen planus is characterized by marked epithelial detachment (16). A distinguishing feature of Esophageal Lichen Planus (ELP) is that it more commonly involves the proximal esophagus and PH studies done for distinguishing it from ulcerative reflux esophagitis are normal. ELP can be distinguished from PV from other physical exam findings such as the involvement of the flexor skin surfaces in PV or the classical finding of a ‘lacelike whitening of the buccal mucosa with ulceration’ in ELP (16).

Treatment of this condition is once again most successful with steroids. Westbrook and Riley in an excellent review of this topic compared studies that used varying treatments for this condition. Oral cyclosporine as well as topical tacrolimus have also been used with success (17).

ELP is a pre-malignant condition, as expected, because of its inflammatory nature. Calabrese and co-workers described the first case of esophageal squamous cell carcinoma associated with ELP in 2003.

In summary, ELP is a rare condition with only 1% of cases of LP involving the esophagus. However, since it is a pre-malignant situation, gastroenterologists should elicit a dermatologic history as well to aid in correctly diagnosing it.

### 3) Eosinophillic Esophagitis (EE)

The differential diagnosis of sloughing esophagitis includes a condition that has only recently been described in literature. EE is a condition that involves eosinophillic influx into the esophageal epithelium. It affects both children and adults. Adults present with dysphagia whereas children with vague abdominal complaints (18). The clinical symptoms as well as pathologic features of EE and gastro esophageal reflux disease (GERD) are similar (19) so biopsy is needed in order to correctly diagnose this condition. However, the clinical presentation in conjunction with the endoscopic findings as well as pathology is crucial to diagnosis since eosinophilia in the esophagus is a non-specific finding. Infections, Crohn’s disease, malignancies, medication, chemotherapy are all associated with eosinophilia (20).

A primary endoscopic difference to note between EE and GERD is that EE often involves long segments of the esophagus and could be patchy/focal and frequently involves the proximal esophagus (21). GERD, however, typically involves the distal much more frequently than the proximal esophagus. Because of the similarity between them, GERD should be excluded by high dose proton pump inhibitor treatment or by evidence of normal pH by esophageal testing, prior to treatment with steroids. More research is needed on this condition, and until further research establishes different diagnostic tests and criteria, clinical and pathological response to therapy is considered to be the absolute confirmation of this diagnosis.

### 4) Medications

**Figure 2:**
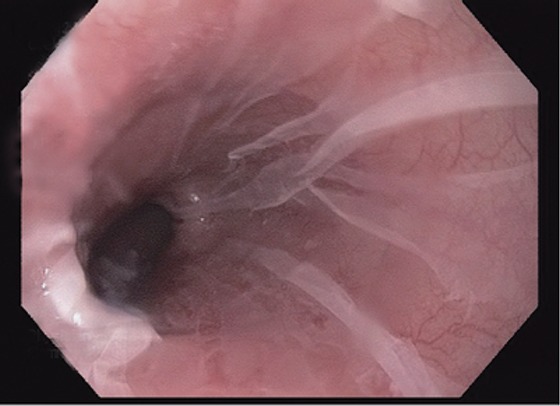
The sloughed squamous epithelium in this case is translucent, with erythematous mucosa visible below. Reproduced with permission from *Modern Pathology* (2012) 25, 767–775; doi: 10.1038/modpathol.2011.204; Julianne K Purdy, Henry D Appelman and Barbara J McKenna.

Medications such as Alendronate and other Bisphosphonates have also been linked to sloughing esophagitis (22) (Fig. 2). Esophageal injury related to bisphosphonate use was reported soon after the approval of bisphosphonates. Non-steroidal Anti-inflammatory drugs (NSAIDS) are one of the more common causes of gastritis and esophagitis. Bisphosphonate use in conjunction with NSAIDs is synergistic in causing gastric ulcers and esophagitis. It is best to prevent their concurrent usage.

### 5) Celiac disease

Case reports have associated sloughing esophagitis with celiac disease (23). Celiac disease has a dermatologic manifestation known as dermatitis herpetiformis and the etiology is known to involve IgA deposition at the dermal-epidermal junction (24). Biopsies from the esophagus in the patient with celiac disease showed pathology similar to that obtained from skin biopsies in patients with dermatitis herpetiformis. Moreover, the esophageal symptoms improved after resumption of a gluten-free diet suggesting an etiologic link (25).

### 6) Corrosive esophagitis

Hot beverages and chemical irritants are other causes of this entity. Ingestion of corrosive substances occurs in more than 26,000 patients a year in US. About 17,000 are children. They are mostly suicide attempt in adults and accidental ingestion in children. Half of ingestions injure the esophagus severely enough to cause long term sequels. Squamous cell cancer of the esophagus is 1000 times more common in these patients so yearly endoscopic surveillance beginning 20 years after injury is recommended.

Alkaline substances produce a deeper depth of injury than acidic substances. Liquefaction necrosis and eschar formation that happens with acids in the superficial esophagus protects deeper layers of esophagus from injury.

Diagnosis is by history but endoscopy might be needed for confirmation. As for treatment there is no role for inducing emesis, lavage or using a neutralization agent because of the chance of further mucosal damage. Corticosteroids have reduced stricture formation from 41% to 19% but only in second and third degree burns.

### 7) Thymoma

In 1997, in a study on chronic sloughing esophagitis, Ponsot and coworkers reported that 2 out of 5 patients had thymoma (4).

## CONCLUSION

Sloughing esophagitis is a rare and underdiagnosed entity. Many times it is seen in debilitated patients on several medicines. If associated with bullous dermatoses, treatment with steroids is usually warranted and effective.
